# Supplementary material for: Unhygienic stool-disposal practices among mothers of children under five in Cambodia: Evidence from a demographic and health survey
Source: PLoS One. 2021 Jul 1;16(7):e0249006. doi: 10.1371/journal.pone.0249006 (PMC8248716; doi:10.1371/journal.pone.0249006)
Supplement: Attachment — Submitted filename: PONE-D-20-17725_reviewer (1).pdf [file pone.0249006.s001.pdf]

PLOS ONE

Unhygienic stool-disposal practices among children under five in Cambodia: evidence from a demographic and health survey

--Manuscript Draft--

**Manuscript Number:**	PONE-D-20-17725
**Article Type:**	Research Article
**Full Title:**	Unhygienic stool-disposal practices among children under five in Cambodia: evidence from a demographic and health survey
**Short Title:**	Unhygienic stool-disposal practices among children under five in Cambodia
**Corresponding Author:**	Pisey Vong<br>Office of Rural Health Care, Pursat Provincial Department of Rural Development<br>Kampong Cham province, Kampong Cham province CAMBODIA
**Keywords:**	Disposal of children's stools, children under five, svyset, Cambodia
**Abstract:**	Abstract<br>Background: Unhygienic disposal of children's stools affects children's health in terms of their susceptibility to many diseases. However, there are no existing studies into the impact of unhygienic stool disposal in Cambodia. Therefore, this study aimed to identify factors associated with the unhygienic disposal of children's stools among children under the age of five in Cambodia.<br>Methods: An analytical cross-sectional study was conducted using data from the Cambodia Demographic and Health Survey (CDHS) 2014. A multivariate binary logistic regression was conducted using the Stata command svyset to analyze factors associated with the unhygienic disposal of children's stools.<br>Results: Overall, the prevalence of practicing unhygienic disposal of children's stools was 29.27% (95%CI: 27.51%- 31.09%). Factors statistically associated with this practice were: region (namely Central Plain, Plateau and Mountains, Coastal and Sea regions), poor household wealth, the mother having no education, a high number of children aged under five, being in the "other" religious category, living in a household with unimproved toilet facilities, living in a household where adequate hygiene is not practiced, and the household not being visited by family planning worker in the last year. However, an increase in the child's age by even a month had significant negative associations with unhygienic practice, even when controlling for other covariates.<br>Conclusion: Almost one third of the mothers do not practice hygienic disposal of children's stools in Cambodia. Unhygienic practices were more prevalent in certain regions, and were also associated with low wealth, lack of education, an increase in the number of children under five in the household, religion, lack of sanitation and access to healthcare professionals. Conversely, the child's age was found to be positively associated with the hygienic disposal of children's stools.
**Order of Authors:**	Pisey Vong
	Samphors Sim
	Rebecca S Dewey
**Additional Information:**	
**Question**	**Response**
**Financial Disclosure**<br><br>Enter a financial disclosure statement that describes the sources of funding for the work included in this submission. Review the submission guidelines for detailed requirements. View published research articles from *PLOS ONE* for specific examples.	The author(s) received no specific funding for this work

This statement is required for submission and **will appear in the published article** if the submission is accepted. Please make sure it is accurate.<br><br>**Unfunded studies**<br>Enter: *The author(s) received no specific funding for this work.*<br><br>**Funded studies**<br>Enter a statement with the following details:<br>• Initials of the authors who received each award<br>• Grant numbers awarded to each author<br>• The full name of each funder<br>• URL of each funder website<br>• Did the sponsors or funders play any role in the study design, data collection and analysis, decision to publish, or preparation of the manuscript?<br>• **NO** - Include this sentence at the end of your statement: *The funders had no role in study design, data collection and analysis, decision to publish, or preparation of the manuscript.*<br>• **YES** - Specify the role(s) played.<br><br>* typeset	
**Competing Interests**<br><br>Use the instructions below to enter a competing interest statement for this submission. On behalf of all authors, disclose any competing interests that could be perceived to bias this work—acknowledging all financial support and any other relevant financial or non-financial competing interests.<br><br>This statement **will appear in the published article** if the submission is accepted. Please make sure it is accurate. View published research articles from *PLOS ONE* for specific examples.	The authors have declared that no competing interests exist

Powered by Editorial Manager® and ProduXion Manager® from Aries Systems Corporation

**NO authors have competing interests**<br><br>Enter: *The authors have declared that no competing interests exist.*<br><br>**Authors with competing interests**<br><br>Enter competing interest details beginning with this statement:<br><br>*I have read the journal's policy and the authors of this manuscript have the following competing interests: [insert competing interests here]*<br><br>* typeset	
**Ethics Statement**<br><br>Enter an ethics statement for this submission. This statement is required if the study involved:<br><br>• Human participants<br>• Human specimens or tissue<br>• Vertebrate animals or cephalopods<br>• Vertebrate embryos or tissues<br>• Field research<br><br>Write "N/A" if the submission does not require an ethics statement.<br><br>General guidance is provided below. Consult the submission guidelines for detailed instructions. **Make sure that all information entered here is included in the Methods section of the manuscript.**	Data from the CDHS were used after having obtained permission. The research project was conducted following additional approval received from the Khon Kean University Ethics Committee in Human Research (Ref: HE632097).

Powered by Editorial Manager® and ProduXion Manager® from Aries Systems Corporation

**Format for specific study types**<br><br>**Human Subject Research (involving human participants and/or tissue)**<br>• Give the name of the institutional review board or ethics committee that approved the study<br>• Include the approval number and/or a statement indicating approval of this research<br>• Indicate the form of consent obtained (written/oral) or the reason that consent was not obtained (e.g. the data were analyzed anonymously)<br><br>**Animal Research (involving vertebrate animals, embryos or tissues)**<br>• Provide the name of the Institutional Animal Care and Use Committee (IACUC) or other relevant ethics board that reviewed the study protocol, and indicate whether they approved this research or granted a formal waiver of ethical approval<br>• Include an approval number if one was obtained<br>• If the study involved *non-human primates*, add *additional details* about animal welfare and steps taken to ameliorate suffering<br>• If anesthesia, euthanasia, or any kind of animal sacrifice is part of the study, include briefly which substances and/or methods were applied<br><br>**Field Research**<br><br>Include the following details if this study involves the collection of plant, animal, or other materials from a natural setting:<br>• Field permit number<br>• Name of the institution or relevant body that granted permission	
**Data Availability**<br><br>Authors are required to make all data underlying the findings described fully available, without restriction, and from the time of publication. PLOS allows rare exceptions to address legal and ethical concerns. See the PLOS Data Policy and FAQ for detailed information.	Yes - all data are fully available without restriction

Powered by Editorial Manager® and ProduXion Manager® from Aries Systems Corporation

A Data Availability Statement describing where the data can be found is required at submission. Your answers to this question constitute the Data Availability Statement and **will be published in the article**, if accepted.<br><br>**Important:** Stating 'data available on request from the author' is not sufficient. If your data are only available upon request, select 'No' for the first question and explain your exceptional situation in the text box.<br><br>Do the authors confirm that all data underlying the findings described in their manuscript are fully available without restriction?	
**Describe where the data may be found in full sentences. If you are copying our sample text, replace any instances of XXX with the appropriate details.**<br><br>• If the data are **held or will be held in a public repository**, include URLs, accession numbers or DOIs. If this information will only be available after acceptance, indicate this by ticking the box below. For example: *All XXX files are available from the XXX database (accession number(s) XXX, XXX.*).<br>• If the data are all contained **within the manuscript and/or Supporting Information files**, enter the following: *All relevant data are within the manuscript and its Supporting Information files.*<br>• If neither of these applies but you are able to provide **details of access elsewhere**, with or without limitations, please do so. For example:<br><br>*Data cannot be shared publicly because of [XXX]. Data are available from the XXX Institutional Data Access / Ethics Committee (contact via XXX) for researchers who meet the criteria for access to confidential data.*<br><br>*The data underlying the results presented in the study are available from (include the name of the third party*	Data can be downloaded from the DHS Data Archive after user registration and getting approval for dataset access. The DHS Programme, Demographic and Health Surveys, Cambodia 2014: https://dhsprogram.com/data/Access-Instructions.cfm

and contact information or URL).<br>• This text is appropriate if the data are owned by a third party and authors do not have permission to share the data.<br><br>* typeset	
Additional data availability information:	Tick here if the URLs/accession numbers/DOIs will be available only after acceptance of the manuscript for publication so that we can ensure their inclusion before publication.; Tick here if your circumstances are not covered by the questions above and you need the journal's help to make your data available.

Powered by Editorial Manager® and ProduXion Manager® from Aries Systems Corporation

Unhygienic stool-disposal practices among children under five in Cambodia: evidence from a demographic and health survey

Vong Pisey[1*], Sim Samphors[2], Rebecca S Dewey [3]

[1]Office of Rural Health Care, Pursat Provincial Department of Rural Development, Ministry of Rural Development, Cambodia
[2]Chea Sim University of Kamchaymear, Cambodia
3 University of Nottingham, United Kingdom
*Corresponding author: piseyvong11@gmail.com (VP)

Abstract

Background: Unhygienic disposal of children's stools affects children's health in terms of their susceptibility to many diseases. However, there are no existing studies into the impact of unhygienic stool disposal in Cambodia. Therefore, this study aimed to identify factors associated with the unhygienic disposal of children's stools among children under the age of five in Cambodia.

Methods: An analytical cross-sectional study was conducted using data from the Cambodia Demographic and Health Survey (CDHS) 2014. A multivariate binary logistic regression was conducted using the Stata command *svyset* to analyze factors associated with the unhygienic disposal of children's stools.

Results: Overall, the prevalence of practicing unhygienic disposal of children's stools was 29.27% (95%CI: 27.51%- 31.09%). Factors statistically associated with this practice were: region (namely Central Plain, Plateau and Mountains, Coastal and Sea regions), poor household wealth, the mother having no education, a high number of children aged under five, being in the "other" religious category, living in a household with unimproved toilet facilities, living in a household where adequate hygiene is not practiced, and the household not being visited by family planning worker in the last year. However, an increase in the child's age by even a month had significant negative associations with unhygienic practice, even when controlling for other covariates.

Conclusion: Almost one third of the mothers do not practice hygienic disposal of children's stools in Cambodia. Unhygienic practices were more prevalent in certain regions, and were also associated with low wealth, lack of education, an increase in the number of children under five in the household, religion, lack of sanitation and access

to healthcare professionals. Conversely, the child's age was found to be positively associated with the hygienic disposal of children's stools.

Keywords: Disposal of children's stools, children under five, svyset, Cambodia.

Introduction

Globally, 2.4 billion people have no access to improved sanitation facilities. One in eight people worldwide (a total of 946 million people) practiced open defecation (OD) in 2015 (1), which decreased to 673 million in 2017 (2). OD can cause both acute and chronic health effects. Acute effects include infectious intestinal disease including diarrheal disease, adverse pregnancy outcomes, and life-threatening violence against women and girls. Chronic effects include soil-transmitted helminthiases, increased anemia, giardiasis, environmental enteropathy and small-intestine bacterial overgrowth, and stunting and long-term impaired cognition (3). In 2015, 47% of the population in Cambodia practiced OD (4). According to the WHO/UNICEF Joint Monitoring Programme (JMP) for Water Supply and Sanitation, a child's feces is classified as being disposed of hygienically when the child passes stools using a toilet or latrine, the feces are put or rinsed into the toilet or latrine, or the feces are buried (5). Unsanitary disposal of children's feces can impact children's health in ways such as environmental enteropathy, impaired growth, and childhood ~~diarrhea~~ (6,7). A study in Cambodia found that unsanitary disposal of children's stool increased the risk of diarrhea by 32% among infants aged 12 to 35 months (7).

Cambodia has made ~~good~~ progress in the provision of improved sanitation facilities, marked by an increase in availability from 3% to 42% between 1990 and 2015. Conversely, this decreased the practice of OD from 89% to 47% in the same period (4). Cambodia's Ministry of Rural Development, (MRD); in collaboration with partners and NGOs (Non-Governmental Organizations) has been implementing the Community-Led Total Sanitation (CLTS) program according to the national guidelines. CLTS has been active in Cambodia since 2005. CLTS is an innovative approach for mobilizing communities to completely eliminate OD. CLTS focuses on the behavioral change needed to ensure real and sustainable improvements (8).

CLTS focuses on hygienic behaviors including the safe disposal of a child's feces, as well as stopping OD, ensuring hygienic toilet use, hygienic practices around food, washing hands at appropriate times; and maintaining a clean and safe environment (9). The Royal Government of Cambodia (RGC) aims to achieve 100% availability of Water and Sanitation by 2025, clearly stating that "everyone must have access to a water supply and live in a hygienic environment by 2025" (10). Therefore, unhygienic disposal children's feces is one of the most important problems to solve.

It is of great importance to understand the factors associated with unhygienic stool disposal practices affecting children under the age of five in Cambodia. At the time of writing, there are no national studies on unhygienic practices around the disposal of children's stools in Cambodia.

Methods

Data source

This research study was an analysis of secondary data from the Cambodia Demographic and Health Survey (CDHS), 2014. The CDHS 2014 is the fourth CDHS targeted to provide information related to fertility, family planning, maternal and child health, gender, HIV/AIDS, malaria, and nutrition. Out of the seven data files available, two relevant data files were included in the study; one containing household data and the other containing children's data. These data were used to explore the factors associated with the unhygienic disposal of children's stools using multiple logistic regression. Further detailed information about the sampling methods used and about CDHS can be found in a previous publication and in the CDHS report (7,11).

Inclusion/exclusion criteria

Data pertaining to practices related to the disposal of children's stools were collected for all children born during the five years preceding the 2014 CDHS. If more than one child aged under five was living in a household with their mother, we included only the youngest child in each household in our analysis.

Outcome variable

The prevalence of unsanitary disposal of children's stools is the outcome variable considered in this study. It is referred to in the questionnaire thus: "The last time (NAME FROM 553) passed stools, what was done to dispose of the stools?" According to the WHO/UNICEF definition (5), we generated and recoded a new binary variable: the child using a toilet or latrine, disposing of or rinsing the child's stool into a toilet or latrine, or burying the child's stool were all coded as hygienic disposal of children's stool and assigned the value "0". Conversely, disposing of or rinsing the child's stool into a drain or ditch, throwing it into garbage, leaving it in the open or not disposing of at all were assigned the code "1".

Explanatory variables

Explanatory variables used were geographical region, residence, wealth index, maternal and paternal education, child's age, mother's age, number of children aged 5 and under in the household, religion, parental marital status, drinking water source, and whether or not the same source was used during dry and wet seasons, type of toilet facility, use of hygiene, exposure to media, and access to family planning and health professionals. Geographical regions were (12) *Tonle Sap*; *Central Plain*; *Plateau and Mountains*; and *Coastal and Sea*. The residence was classified as either *urban* or *rural*. Wealth index was recoded as a binary variable, with those in the poorest and poorer quintiles both coded as *poor*, and those in middle, richer and richest quintiles coded as *non-poor* (13). Mother's education was recoded as a binary variable, with *no education* retaining the classification, and those receiving primary, secondary and higher levels of education were all recoded as having had *education* (14). Similarly, the father's education was recoded as binary variable; *no education* retaining its coding unchanged, and those receiving primary, secondary and higher were recoded as having had *education*. The *child's age* was treated as a continuous variable. The *mother's age* was coded as a discrete variable with three coded values; *15-24 years*, *25-34 years* and ≥ *35 years*). The *number of children aged 5 and under* in the household was treated as a continuous variable. Religion was recoded as binary variable, *Buddhist* and *non-Buddhist* (Muslim, Christian, and other religions were

recoded as other religion). Maternal marital status was recoded as a binary variable; married and living with partner were coded as *living with partner*, whereas widowed, divorced, and no longer living together/separated were recoded as *separated*. The source of drinking water used by the household during the dry season was recoded as either *improved* or *unimproved* (15). Similarly, the source of drinking water during wet season was recoded as either *improved* or *unimproved* (15). Use of the same or different sources of drinking water during both wet and dry seasons was coded as either *same* or *different*. The type of toilet facility used by members of the household was recoded as either *improved* or *unimproved* (15). Whether or not adequate hygiene was used in the household was recoded as a binary variable; *using adequate hygiene* and *not using adequate hygiene* (15). Exposure to media was recoded as a binary variable; *exposed* and *not exposed* (16). The occurrence of visits by family planning health workers in the last year was coded as either *yes* or *no*. Similarly, whether or not they had visited a health institution in the last year was coded as *yes* or *no*. The children's experience of diarrhea in the last two weeks was coded as a binary variable; those responding no and don't know were recoded as *no*, and those responding yes retained the coding of *yes*.

Data use

Data from the CDHS 2014 were used in this study.

Statistical analysis

The analysis was performed using STATA/SE version 14.0. The women's individual sample weightings were used in the estimation. Frequency analysis of categorical data was performed to provide number and percentage. Continuous data was analysed as means, standard deviations, and ranges. Cross-tabulations were performed with the individual sample weightings to provide nationally representative results (17). Bivariate and multivariate binary analyses were used to assess the effects and interactions of associated factors on the unhygienic disposal of children's stools. An F-test was used in the bivariate analysis to determine the association between the outcome and the explanatory variables. A linearity test was conducted between the

continuous variable and outcome variable. Any explanatory variables significant at $p<0.25$ were entered into the initial model (18,19). Multi-collinearity of the outcome variables was assessed by excluding those with a variance inflation factor (VIF) greater than 4. Multivariate binary logistic regression was used to find the factors associated with the unhygienic disposal of children's stools. The *svyset* command was used to test for complex survey sampling methods used in the original surveys, in order to adjust for differences in the probabilities of sample selection, and to avoid using over-sampled strata within the survey data (20). Differences associated with factors are reported using the adjusted odds ratio (AOR).

Ethics

Data from the CDHS were used after having obtained permission. The research project was conducted following additional approval received from the Khon Kean University Ethics Committee in Human Research (Ref: HE632097).

Results

Among the 5,745 households in the sample, 45% live in the Central Plain region. The majority (85.77%) live in rural areas. 43.35% classified as poor wealth. The majority of parents (86.58% of mothers and 90.62% for fathers) had received education. The average child age was 26.16±16.94 months, and the average maternal age was 28.76±6.10 years. The majority of the sample (95.64%) were Buddhist. 62.07% of the sample used an improved drinking water supply during dry season while 82.45% used an improved drinking water supply during wet season. 41.86% of the sample used improved toilet facilities and 66.93% used adequate hygiene. 25.65% had been visited by a family planning health worker in the last year, whereas 59.17% had visited a healthcare facility in the last year. 13.54% of children had experienced diarrhea in the last two weeks (Table 1).

Bivariate analysis of factors associated with unhygienic stool disposal among children under five in Cambodia

Table 2 shows the factors that had statistically significant associations with unhygienic stool disposal among children under five ($p<0.05$); these were region, wealth index, maternal and paternal education, the child's age, the mother's age, the number of children in the household aged under five, the religion, the source of drinking water during dry season, whether or not the same source of drinking water was used during wet and dry season, the toilet facility, use of hygiene, exposure to the media, whether or not the family had been visited by a family planning health worker in the last year, and whether or not the child had experienced diarrhea in the last two weeks. The remaining factors of the source of drinking water used during the wet season, and whether or not the family had visited a healthcare institution in the last year did not reach significance, however they did reach the pre-determined threshold of $p<0.25$ for inclusion in the initial model.

Multivariate analysis of factors associated with unhygienic stool disposal among the under-fives in Cambodia

Table 3 shows that households in the Central Plain region, Plateau and Mountains region, and Coastal and Sea region had higher odds ([AOR = 1.65; 95% CI: 1.33-2.04], [AOR = 2.53; 95% CI: 1.98-3.24], and [AOR = 4.16; 95% CI: 3.15-5.48], respectively)) of practicing the unhygienic disposal of children's stools than those in Tonle Sap region. The odds of practicing the unhygienic disposal of children's stools were 58% higher in households with poor wealth compared to non-poor, and were 45% higher in mothers who had no education compared to those having education. As the child's age increased by a month, the odds of unhygienic stool disposal decreased by 35%. As the number of children under the age of five in the household increased by one, the odds of unhygienic stool disposal increased by 11%. The odds of practicing unhygienic disposal of children's stools was 77% higher in non-Buddhist households compared to Buddhist households. The odds were 22% higher in households with unimproved toilet facilities compared with those with improved toilet facilities. The odds were 33% higher in households using inadequate hygiene compared with those using adequate hygiene. The odds of practice unhygienic

disposal of children's stools were 45% higher in households that had not been visited by a family planning health worker in the last year compared with those were visited.

Table 1. Characteristics of households in Cambodia, 2014 (n=5,745).

**Variables**	**Number**	**Percentage**
**Region**		
Tonle Sap	1846	32.13
Central Plain	2605	45.34
Plateau and Mountains	933	16.24
Coastal and Sea	361	6.29
**Residence**		
Urban	817	14.23
Rural	4928	85.77
**Wealth index**		
Non-poor	3255	56.65
Poor	2490	43.35
**Maternal Education**		
Had education	4974	86.58
No education	771	13.42
**Paternal Education**		
Had education	5207	90.62
No education	538	9.38
**Child's age (months)**		
48-59	863	15.04
36-47	940	16.37
24-35	1136	19.77
12-23	1368	23.80
<12	1438	25.03
Mean±SD	26.16±16.94	
Range	0 to 59	
**Mother's age (years)**		
≥35	925	16.11
25-34	3232	56.25
15-24	1588	27.64
Mean±SD	28.76±6.10	
Range	15 to 49	
**Number of children under five years**		
1	3812	66.35
2-6	1933	33.65
Mean±SD	1.41±0.64	
Range	1 to 6	
**Religion**		
Buddhism	5495	95.64
Other	250	4.36
**Mother's marital status**		

Variables	Number	Percentage
Separated	263	4.58
Living with partner	5482	95.42
**Source of drinking water during dry season**		
Improved	3566	62.07
Unimproved	2179	37.93
**Source of drinking water during wet season**		
Improved	4737	82.45
Unimproved	1008	17.55
**Same source during wet and dry seasons**		
Same	3967	69.05
Different	1778	30.95
**Toilet facility**		
Improved	2405	41.86
Unimproved	3340	58.14
**Hygiene**		
Adequate	3845	66.93
Inadequate	1900	33.07
**Exposure to media**		
Yes	3637	63.30
No	2108	36.70
**Family planning health worker visit in last year**		
Yes	1473	25.65
No	4272	74.35
**Visited healthcare institution in last year**		
No	2346	40.83
Yes	3399	59.17
**Children with diarrhea in last two weeks**		
No	4968	86.46
Yes	777	13.54

Table 2: Bivariate analysis of factors associated with unhygienic stool disposal (USD) of children under the age of five in Cambodia, 2014 (n=5,745).

Variables	Number	USD %	COR	95% CI	p-value
**Overall**	5745	29.27		27.51-31.09	
**Region**					<0.001
Tonle Sap	1846	21.61	1		
Central Plain	2605	27.79	1.40	1.14-1.71	
Plateau and Mountains	933	41.53	2.58	2.01-3.30	
Coastal and Sea	361	47.35	3.26	2.46-4.34	
**Residence**					0.630
Urban	817	28.47	1		
Rural	4928	29.40	1.05	0.87-1.26	

Variables	Number	USD %	COR	95% CI	p-value
**Wealth index**					<0.001
Non-poor	3255	22.78	1		
Poor	2490	37.74	2.05	1.74-2.42	
**Maternal Education**					<0.001
Had education	4974	27.57	1		
No education	771	40.20	1.77	1.40-2.23	
**Paternal Education**					<0.001
Had education	5207	28.31	1		
No education	538	38.54	1.59	1.22-2.06	
**Child's age (months)**	5745	N/A	0.66	0.62-0.71	<0.001
**Mother's age (years)**					
≥35	925	25.84	1		0.005
25-34	3232	28.43	1.14	0.92-1.41	
15-24	1588	32.96	1.41	1.12-1.79	
**Number of children under five years**	5745	N/A	1.24	1.17-1.33	<0.001
**Religion**					<0.001
Buddhism	5495	28.33	1		
Other	250	49.80	2.51	1.65-3.81	
**Mother's marital status**					0.252
Separated	263	25.56	1		
Live with partner	5482	29.44	1.22	0.87-1.70	
**Source of drinking water during dry season**					0.003
Improved	3566	27.29	1		
Unimproved	2179	32.49	1.28	1.08-1.52	
**Source of drinking water during wet season**					0.066
Improved	4737	28.48	1		
Unimproved	1008	32.96	1.23	0.99-1.55	
**Same source during wet and dry season**					0.004
Same	3967	27.71	1		
Different	1778	32.73	1.27	1.08-1.50	
**Toilet facility**					<0.001
Improved	2405	21.25	1		
Unimproved	3340	35.03	1.41	1.30-1.54	
**Hygiene**					<0.001
Adequate	3845	25.81	1		
Inadequate	1900	36.27	1.64	1.37-1.95	
**Exposure to media**					<0.001
Yes	3637	26.06	1		
No	2108	34.80	1.51	1.29-1.77	
**Family planning health**					0.002

Variables	Number	USD %	COR	95% CI	p-value
**worker visit in last year**					
Yes	1473	25.14	1		
No	4272	30.69	1.32	1.10-1.58	
**Visited healthcare institution in the last year**					0.051
No	2346	27.33	1		
Yes	3399	30.60	1.17	0.99-1.37	
**Children with diarrhea in the last two weeks**					<0.001
No	4968	28.16	1		
Yes	777	36.36	1.46	1.18-1.80	

Table 3: Multivariate analysis of factors associated with unhygienic stool disposal (USD) of children under the age of five in Cambodia, (n= 5,745)

Variables	Number	USD %	AOR	95% CI	p-value
**Region**					<0.001
Tonle Sap	1846	21.61	1		
Central Plain	2605	27.79	1.65	1.33-2.04	
Plateau and Mountains	933	41.53	2.53	1.98-3.24	
Coastal and Sea	361	47.35	4.16	3.15-5.48	
**Wealth index**					<0.001
Non-poor	3255	22.78	1		
Poor	2490	37.74	1.58	1.31-1.91	
**Maternal Education**					0.003
Had education	4974	27.57	1		
No education	771	40.20	1.45	1.14-1.85	
**Child's age (months)**	5745	N/A	0.65	0.60-0.70	<0.001
**Number of children under five years**	5745	N/A	1.11	1.03-1.20	<0.001
**Religion**					0.001
Buddhism	5495	28.33	1		
Other	250	49.80	1.77	1.25-2.51	
**Toilet facility**					<0.001
Improved	2405	21.25	1		
Unimproved	3340	35.03	1.22	1.11-1.34	
**Hygiene**					<0.001
Adequate	3845	25.81	1		
Inadequate	1900	36.27	1.33	1.12-1.59	
**Family planning health**					<0.001

Variables	Number	USD %	AOR	95% CI	p-value
**worker visit in last year**					
Yes	1473	25.14	1		
No	4272	30.69	1.45	1.19-1.77	

Discussion

Alarmingly, our study has shown that as many as one third of Cambodian mothers or caregivers of children practiced unhygienic stool disposal. However, this prevalence is not dissimilar to that found in a previous study conducted in Madagascar (38%) (21). Furthermore, unhygienic practices are much less prevalent in Cambodia in comparison to Zambia, Kenya, Uganda and Malawi, with prevalence rates of 67%, 70%, 75%, and 79% respectively (22–25). Conversely, other studies have found even higher prevalence rates (81.4%) in India and lower again in Ethiopia (38%) (26). In our recent study, many associated factors have been found to be associated with unhygienic stool disposal practices: geographical region (with Central Plain region, Plateau and Mountains region, Coastal and Sea region being worst), poor household wealth, lack of maternal education, a high number of children under five children in the household, non-Buddhist religion, use of unimproved toilet facilities, adequate use of hygiene, and not being visited by a family planning worker in the last year. However, an increase in the child's age by as little as a month had a significant negative associations with the unhygienic disposal of children's stools, when controlling for other covariates.

The region was significantly associated with unhygienic stool disposal, explicitly that the Coastal and Sea region, Central Plain, and Plateau and Mountains exhibited AORs 4.16 times, 2.53 times, and 1.65 times of that in the Tonle Sap lake region. This is in agreement with a study from Kenya which found that mothers and caregivers who are living in urban areas had better understanding and awareness of sanitation and hygiene (27). Therefore, households in the Coastal and Sea region are most at risk; it might be that the mothers and caregivers in this region have not received adequate information or intervention with regards to sanitation, due to the distance of the region

from the city. Therefore, households in the rural areas of the region may be strongly impacted the practice of unhygienic stool disposal.

Wealth index was also significantly associated with unhygienic stool disposal, with mothers and caregivers living in poverty being 1.58 times (AOR 1.58, 95% CI 1.31-1.91) more likely to have unhygienic practices compared to those not in poverty. This finding is consistent with a study from India (28) which showed that the household earning ≤23.9 USD per month is less likely by a factor of 4.7 to have hygienic practices compared to those who earned >23.9 USD. Moreover, a study from Bangladesh (29) has shown that households with high socioeconomic status are less likely to practice unhygienic stool disposal compared to low socioeconomic status households (30). It is likely that the mothers and caregivers in wealthier households experience a better standard of living and have more knowledge related to the hygienic disposal of children's stools. Further, a status of higher wealth will allow the mothers and caregivers to improve their standard of living.

The level of the mother's education plays an important role in the hygienic disposal of children's stools. This study found that a mother who had no education was more likely (AOR 1.45; 95%CI: 1.14-1.85) to practice unhygienic stool disposal compared to a mother who had received formal education. This is in agreement with the findings of the National Family Health Survey (NFHS-3 2005-2006), in which unhygienic behaviors were 61% more likely in mothers who had finished secondary school compared to mothers who had not (31). Similarly, a study from Accra found that mothers who had finished primary school were more likely to practice healthy stool disposal (32) than those who had not. Specifically, mothers who had awareness and understanding about the causes and impacts of unhygienic stool disposal were able to practice well compared to mothers who had no or a lower level of education (33). It can be seen that education is absolutely vital for mothers and caregivers who are looking after children since they need knowledge about the root causes of problems and the impacts of their behaviors on the child's health.

An increase in the child's age by as little as a month had a significant, negative association with the practice of unhygienic disposal of children's stools. This is in line with a study conducted in India and Cambodia (14,34) in which the older the child, the safer the practice of stool disposal. Additionally, our findings are that as the

number of children under the age of five increased, the risk of unsafe disposal of the children's stool increased, again in line with a study in Ethiopia (35).

Importantly, the present study found that religion was significantly associated with unhygienic stool disposal practices. Mothers who were not Buddhist were 1.77 times more likely to practice unhygienic stool disposal compared to mothers who were Buddhist. This finding is consistent with a study in Ethiopia that found religion to be a key factor, with mothers who were Catholic, Protestant and Muslim being 3.15 times, 1.64 times and 1.02 times more likely to have poor hygiene, and mothers with traditional beliefs to be 0.74 times as likely, as compared to Buddhist mothers (35). This finding may be related to the practice in Buddhism of monks playing an important role during ceremonies in giving out advice to the people in the community, especially that related to hygiene and sanitation. Hence, followers of Buddhism have more time to engage in informal education from Buddhist monks.

Most importantly, the quality of the toilet facility is significantly associated with unhygienic stool disposal practices. Households that had improved toilet facilities were more likely to practice hygienic stool disposal (AOR: 1.22) compared to households where the toilet facility had not been improved. This result is in line with studies in India and rural Bangladesh (14,36). Moreover, this finding is consistent with studies reporting that the households failing to follow hygienic stool disposal practices had not improved their toilet (22,37). Similarly, in Ethiopia and South Africa, improvement of households' toilet facilities correlated highly with the likelihood of practicing hygienic stool disposal (23,25). Therefore, sanitation interventions should not neglect the importance of behavioral changes. Latrine improvement is crucial to ensure that mothers and caregivers do not face additional risks resulting from their standard of living.

We also found inadequate use of hygiene generally was significantly associated with unhygienic stool disposal. Mothers who had inadequate hygiene were more likely (AOR = 1.33) to dispose of their child's stool unhygienically compared to mothers with adequate hygiene. This is consistent with a report from Ethiopia showing that mothers who used unimproved water sources were more likely to practice unhygienic stool disposal practices compared to mothers who favored piped water and water from other improved sources (35). This finding further in line with the findings of a study

conducted in Northern Ethiopia, which revealed that mothers who had used cleaning materials to wash their hands after using the toilet were more likely to practice unhygienic stool disposal compared to mothers using either water only or water and soap. It seems that unhygienic stool disposal is highly related to the mother's broader understanding of what good hygiene involves; further that as the knowledge and awareness of the mother improves, they are less likely to have unhealthy behaviors. Therefore, teaching the understanding of hygiene is paramount for improving quality of life.

Finally, not receiving a visit from a family planning health worker in the last year was significantly associated with unhygienic stool disposal. This finding is in agreement with those from the Ethiopian study (35) that found mothers who had not visited family planning health workers in the last year to be 1.50 times more likely to practice unhygienic stool disposal compared to mothers who had had contact with the family planning health worker in the last year. Receiving advice from family planning health workers is vital for wellbeing, especially for the mothers and caregivers who look after children because this advice provides be the awareness and knowledge necessary for disease prevention.

Acknowledgements

The authors would like to express sincere thanks and appreciation to:

Dr. Kavin Thinkhamrop, Health and Epidemiology Geoinformatics Research (HEGER), Faculty of Public Health, Khon Kaen University; and Dr. Wilaiphorn Thinkhamrop, Data Management and Statistical Analysis Center (DAMASAC), Faculty of Public Health, Khon Kaen University for their statistical support.

REFERENCES

1. World Health Organization. Key facts from 2015 JMP report. 2015.
2. World Health Organization. Progress on household drinking water, sanitation and hygiene 2000-2017: special focus on inequalities. World Health Organization; 2019.
3. Mara D. The elimination of open defecation and its adverse health effects: A

moral imperative for governments and development professionals. J Water Sanit Hyg Dev. 2017;7(1):1–12.
4. World Health Organization, United Nations Children's Fund (UNICEF). Progress on sanitation and drinking water – 2015 update and MDG assessment. World Health Organization; 2015.
5. United Nations Children's Fund (UNICEF), World Health Organization (WHO). Core questions on drinking-water and sanitation for household surveys. World Health Organization. 2006.
6. George CM, Oldja L, Perin J, Sack RB, Biswas S, Ahmed S, et al. Unsafe Child Feces Disposal is Associated with Environmental Enteropathy and Impaired Growth. J Pediatr. 2016;176:43–9.
7. Pisey V, Banchonhattakit P. Influence of socio-demographic and environmental factors on childhood diarrhea in Cambodia. F1000Research. 2020 Apr 28;9(303):303.
8. Ministry of Rural Development. National guidelines on community-led total sanitation (CLTS). Phnom Penh; 2017.
9. Kar K, Chambers R. Handbook on Community-Led Total Sanitation. Vol. 44, Children. 2008. 96 p.
10. Ministry of Rural Development. National Strategy for Rural Water Supply, Sanitation and Hygiene 2011-2025. Phnom Penh; 2011. 109 p.
11. Kosal S, Satia C, Kheam T, Chinda P, Mondol L, Phirun L, et al. Cambodia Demographic and Health Survey 2014. Phnom Penh: National Institute of Statistics, Directorate General for Health, and ICF International. 2015;
12. Ministry of Planning. General Population Census of the Kingdom of Cambodia 2019. 2019.
13. He Z, Bishwajit G, Zou D, Yaya S, Cheng Z, Zhou Y. Burden of common childhood diseases in relation to improved water, sanitation, and hygiene (WASH) among Nigerian children. Int J Environ Res Public Health. 2018;15(6).
14. Bawankule R, Singh A, Kumar K, Pedgaonkar S. Disposal of children's stools and its association with childhood diarrhea in India. BMC Public Health. 2017;17(1):1–9.
15. United Nations Children's Fund (UNICEF), World Health Organization. Core questions on drinking water, sanitation and hygiene for household surveys: 2018 update. New York; 2018.
16. Westoff CF, Bankole A. Mass media and reproductive behaviour in Africa. DHS Analytical Reports no. 2. 1997.
17. Rutstein SO, Staveteig S, Winter R, Yourkavitch J. Urban Child Poverty, Health, and Survival in Low- and Middle-Income Countries. DHS Comparative Reports No. 40. Rockville, Maryland, USA; 2016.
18. Hosmer DW, Lemeshow S. Applied Logistic Regression. 2000. 1-369 p.
19. Hosmer DW, Lemeshow S, Sturdivant RX. Applied Logistic Regression Analysis. The Statistician. New York, United States: John Wiley & Sons Inc; 2013. 528 p.
20. Croft, Trevor N., Aileen M. J. Marshall CKA. Guide to DHS Statistics. Rockville, Maryland, USA ICF. 2018;22–51.
21. Unicef, WSP, World Bank. Child Feces Disposal in Madagascar.

2015;(December 2014):1–4.

22. Unicef, WSP, World Bank. Child Feces Disposal in Zambia. 2015;(December 2014):1–4.
23. Unicef, WSP, World Bank. Child Feces Disposal in Kenya. 2015;(December 2014):1–4.
24. Unicef, WSP, World Bank. Child Feces Disposal in Uganda. 2015;(December 2014):1–4.
25. Unicef, WSP, World Bank. Child Feces Disposal in Malawi. 2015;(December 2014):1–4.
26. National Population Commission (NPC) [Nigeria], ICF International. Nigeria Demographic and Health Survey 2013. Abuja, Nigeria, and Rockville, Maryland, USA; 2014.
27. Kenya National Bureau of Statistics (KNBS), ICF Macro. Kenya Demographic and Health Survey 2008-09. Calverton, Maryland; 2010.
28. Preeti PS, Sahoo SK, Biswas D, Dasgupta A. Unsafe disposal of child faeces: A community-based study in a rural block in West Bengal, India. J Prev Med Public Heal. 2016;49(5):323–8.
29. Begum HA, Moneesha SS, Sayem AM. Child care hygiene practices of women migrating from rural to Urban areas of Bangladesh. Asia-Pacific J Public Heal. 2013;25(4):345–55.
30. Halder AK, Tronchet C, Akhter S, Bhuiya A, Johnston R, Luby SP. Observed hand cleanliness and other measures of handwashing behavior in rural Bangladesh. BMC Public Health. 2010;10.
31. International Institute for Population Sciences (IIPS), Macro International. National Family Health Survey (NFHS-3), 2005-06: India: Volume I. Mumbai; 2007.
32. Armar-Klemesu M, Ruel MT, Maxwell DG, Levin CE, Morris SS. Poor Maternal Schooling Is the Main Constraint to Good Child Care Practices in Accra. J Nutr. 2000;130(6):1597–607.
33. Mwambete, K.D., and Joseph R. Knowledge and perception of mothers and child caregivers on childhood diarrhea management in Tanzania. Tanzan J Health Res. 2010;12(1):1–9.
34. Miller-Petrie MK, Voigt L, McLennan L, Cairncross S, Jenkins MW. Infant and young child feces management and enabling products for their hygienic collection, transport, and disposal in Cambodia. Am J Trop Med Hyg. 2016;94(2):456–65.
35. Azage M, Haile D. Factors associated with safe child feces disposal practices in Ethiopia: Evidence from demographic and health survey. Arch Public Heal. 2015;73(1):1–9.
36. Islam M, Ercumen A, Ashraf S, Rahman M, Shoab AK, Luby SP, et al. Unsafe disposal of feces of children <3 years among households with latrine access in rural Bangladesh: Association with household characteristics, fly presence and child diarrhea. PLoS One. 2018;13(4):1–13.
37. Unicef, WSP, World Bank. Child Feces Disposal in Nepal. 2015;(February 2015):1–4.
