# Supplementary material for: Unhygienic stool-disposal practices among mothers of children under five in Cambodia: Evidence from a demographic and health survey
Source: PLoS One. 2021 Jul 1;16(7):e0249006. doi: 10.1371/journal.pone.0249006 (PMC8248716; doi:10.1371/journal.pone.0249006)
Supplement: Attachment — Submitted filename: Response to Reviewer 3.docx [file pone.0249006.s002.docx]

Reviewer #3: Here is a list of specific comments. Note: page numbering in reviews and comments is based on ruler applied in Editorial Manager-generated PDF. Line numbering is not available.

1. Page 1, Methods, line 2: I suggest replacing “multivariate” with ‘multivariable’ throughout the manuscript.

Reply: Taken care

2. Page 1, Methods, lines 3: I suggest replacing “the Stata command svyset” with ‘Stata’ because the svyset command only declared the design but did not perform the analyses.

Reply: Taken care

3. Page 1, Results: I suggest including more numeric associations. Maybe consider to list the most important factors because of the word limits.

Reply: Taken care. Overall, the prevalence of practicing unhygienic disposal of children’s stools was 29.27% (95%CI: 27.51%- 31.09%). Factors statistically associated with this practice were: living in the Central Plain, Plateau and Mountains, Coastal or Sea regions (AOR = 1.65; 95% CI: 1.33- 2.04), (AOR = 2.53; 95% CI: 1.98-3.24) and (AOR = 4.16; 95% CI: 3.15-5.48) respectively, poor household wealth (AOR = 1.58; 95% CI: 1.31-1.91), the mother having no education (AOR = 1.45; 95% CI: 1.14-1.85), a high number of children aged under five (AOR = 1.11; 95% CI: 1.03-1.20), being in the "other" religious category (AOR = 1.77; 95% CI: 1.25-2.51), living in a household with unimproved toilet facilities (AOR = 1.22; 95% CI: 1.11-1.34), living in a household with inadequate hygiene (AOR = 1.33; 95% CI: 1.12-1.59), and the household not being visited by a family planning worker in the last year (AOR = 1.45; 95% CI: 1.19-1.77). However, an increase in the child's age by even a month had significant negative associations with unhygienic practice (AOR = 0.65; 95% CI: 0.60-0.70), even when controlling for other covariates. 

4. Page 3, Data Source, lines 4–6: Were the seven data files “fertility”, . . . , and “nutrition” that were mentioned in the previous sentence? If so, I suggest replacing “household data” and “chilren’s data” with the names introduced previously.

Reply: No. The seven data files are Household file (HR), Household members, or persons file (PR), Women’s file (IR), All births file (BR), children born in the 5 years prior to the interview, or kids file (KR), Men’s file (MR), and Couple’s file (CR). Therefore, for this analysis, the author(s) use only two data files out of the seven files. These two data files can give all information about our analysis.

5. Page 3, Data Source, lines 6–8: The sentence, “these data were used ... using multiple logistic regression”, was irrelevant for the Data Source section. I suggest removing it.
Reply: Taken care

6. Page 4, Explanatory Variables, lines 10–15: Can these two sentences, “mother’s . . . ” and “similarly, the father’s . . . ”, be combined as one sentence, ‘mother’s and father’s education . . . ’?
Reply: Taken care

7. Page 4, Explanatory Variables, lines 15–17: I was curious why child’s age and mother’s age were treated differently.

Reply: When the author(s) performed linearity test, the child’s age was linearity with the outcome variable, vice versa the mother’s age was not linearity. Therefore, these two variables were treated differently.

8. Page 5, Statistical Analysis, lines 1–2: The sentence, “the women’s individual sample weightings ...”, seemed duplicated comparing with the third subsequent sentence, “cross-tabulations ...”. If they were different, I suggest clarifying their differences. Otherwise, I suggest combining the two sentences into one.

Reply: Taken care: They were the same. The author(s) agree to combine them into one.

9. Page 5, Statistical Analysis, lines 6–7: I was not sure the implication of “bivariate and multivariate”. Did it mean ‘unadjusted and adjusted’?
Reply: Yes, bivariate refers to unadjusted and multivariable refers to adjusted.

10. Page 5, Statistical Analysis, lines 7–9: The outcome was binary. The F-test and linearity test seemed inappropriate for the binary outcome. Unadjusted logistic regressions can be used to examine associations between the binary outcome and explanatory variables. In addition, please elaborate the linearity test.

Reply: Taken care. The author(s) agrees with the comment from reviewer. The outcome was binary, F-test is not related to this. However, we should test for linearity for every continuous explanatory (independent) variables. For those explanatory variables which are linearity with the outcome, we treated it as continuous; and vice versa for those explanatory variables which are not linearity with the outcome, we treated it as categorical group.

11. Page 6, Statistical Analysis, line 2: Please make it clear that “the initial model” referred to “the multivariable binary logistic regression” in the second subsequent sentence.

Reply: Taken care. Any explanatory variables significant at p<0.25 in the bivariate analysis were entered into the initial model.

12. Page 6, Statistical Analysis, lines 2–4: Shouldn’t the multi-collinearity exist among explanatory variables, not the outcome variables? Please clarify and elaborate further. I did not find any findings regarding the multi-collinearity in the Results section.

Reply: Taken care. Multi-collinearity of the explanatory variables was assessed by excluding those with a variance inflation factor (VIF) greater than 4 and no variable with VIF greater than 4 was found.

Reply: After bivariate analysis, we performed command to find the multi-collinearity; however, we don’t see any multi-collinearity from our analysis; therefore, the author(s) didn’t put it in the Results section.

13. Page 6, Statistical Analysis, line 5: I thought the svyset command was only used to declare the survey data. Shouldn’t be there more commands used for further analyses?
Reply: I don’t understand what the reviewer #3 means in this part whether s/he suggests to delete that? The author (s) use svy command to run the analysis.

14. Page 8, Table 1: I suggest including weighted numbers and percentages along with the unweighted ones.

Reply: We do not agree with the reviewer because of the reasons as above: First, DHS data is needed to be weighted to present national. Second, the unweighted will present with different sample size. Our Table 1 is weighted and its sample size is 5,745. If we are unweighted, the sample size is just only 5,681. Therefore, we have retained our original.

(Note: Table 1 has been renamed to Table 2)

15. Page 9, Table 2: I suspected the numbers in the COR column were unadjusted odds ratios. If so, I suggest replacing “COR” with ‘odds ratio’ and replacing “bivariate analysis’ with ‘unadjusted binary logistic regression’.

Reply: Taken care. We agree with the reviewer. (Note: Table 2 has been renamed to Table 3)
