# Supplementary material for: Unhygienic stool-disposal practices among mothers of children under five in Cambodia: Evidence from a demographic and health survey
Source: PLoS One. 2021 Jul 1;16(7):e0249006. doi: 10.1371/journal.pone.0249006 (PMC8248716; doi:10.1371/journal.pone.0249006)
Supplement: Attachment — Submitted filename: Response to Journal Requirements.docx [file pone.0249006.s003.docx]

Journal Requirements:

Please review your reference list to ensure that it is complete and correct. If you have cited papers that have been retracted, please include the rationale for doing so in the manuscript text, or remove these references and replace them with relevant current references. Any changes to the reference list should be mentioned in the rebuttal letter that accompanies your revised manuscript. If you need to cite a retracted article, indicate the article’s retracted status in the References list and also include a citation and full reference for the retraction notice.

Reply: Taken care: The author(s) replace the current update reference.

7.	Pisey V, Banchonhattakit P. The association of socio-demographic and environmental factors on childhood diarrhea in Cambodia. F1000Research. 2020;9:303.
